# An injectable hydrogel to disrupt neutrophil extracellular traps for treating rheumatoid arthritis

**DOI:** 10.1080/10717544.2023.2173332

**Published:** 2023-02-01

**Authors:** Nan Wang, Jie Ma, Wenxia Song, Chengwu Zhao

**Affiliations:** a Department of Hand and Foot Surgery, The First Hospital of Jilin University, Changchun, China; b Department of Clinical pharmacy, The First Hospital of Jilin University, Changchun, China; c Department of Pathology, The First Hospital of Jilin University, Changchun, China; d Department of Sports Medicine, The First Hospital of Jilin University, Changchun, China

**Keywords:** Rheumatoid arthritis, deoxyribonuclease, neutrophil extracellular traps, anti-inflammation, hydrogel

## Abstract

Rheumatoid arthritis (RA), an autoimmune disease, is characterized by inflammatory cell infiltration that damages cartilage, disrupts bone, and impairs joint function. The therapeutic efficacy of RA treatments with the severely affected side remains unsatisfactory despite current treatment methods that primarily focus on anti-inflammatory activity, largely because of the complicatedly pathological mechanisms. A recently identified mechanism for RA development involves the interaction of RA autoantibodies with various proinflammatory cytokines to facilitate the formation of neutrophil extracellular traps (NETs), which increased inflammatory responses to express inflammatory cytokines and chemokines. Therefore, NETs architecture digestion may inhibit the positive-feedback inflammatory signal pathway and lessen joint damage in RA. In this work, deoxyribonuclease I (DNase) is connected to oxidized hyaluronic acid (OHA) via Schiff base reaction to extend the half-life of DNase. The modification does not influence the DNase activity for plasmid deoxyribonucleic acid hydrolysis and NETs’ architecture disruption. Carboxymethyl chitosan is crosslinked with DNase-functionalised OHA (DHA) to form an injectable, degradable, and biocompatible hydrogel (DHY) to further strengthen the adhesive capability of DHA. Importantly, the collagen-induced arthritis model demonstrates that intra-articular injection of DHY can significantly reduce inflammatory cytokine expression and alleviate RA symptoms, which can be significantly improved by combining methotrexate. Here, a DNase-functionalised hydrogel has been developed for RA treatment by constantly degrading the novel drug target of NETs to decrease inflammatory response in RA.

## Introduction

Rheumatoid arthritis (RA), a systemic autoimmune disease characterized by systemic inflammation, leads to cartilage degradation, bone defects, and has been a threat to global health (McInnes & Schett 2011; Szekanecz et al., 2021). Traditional treatments, including anti-inflammatory drugs, help to decrease inflammation and alleviate pain (Lipsky et al., 2000; Bullock et al., 2018). For instance, routine drugs like non-steroidal drugs improve patient quality of life; however, commonly prescribed agents cause numerous side effects, including gastrointestinal irritation, interstitial lung disease, and osteoporosis (Li et al., 2019). Therefore, research into the precise pathological mechanisms of RA and novel therapeutic approaches is urgently needed for effective inflammation inhibition and side effect avoidance.

Previous studies have demonstrated that neutrophils are key cellular components that infiltrate the synovial tissue and rheumatoid nodules of patients with RA (Wright et al., 2014; 2020). Neutrophils mediate the pathogenesis of arthritis through promoting the inflammatory process and cartilage degradation (Jasin & Taurog 1991). Activated neutrophils with upregulated major histocompatibility complex class II molecule expression can present antigens to T lymphocytes and induce inflammatory cytokine expression, such as interleukin (IL)-1β and tumor necrosis factor (TNF)-α (O’Neil & Kaplan 2019). According to recent evidence, the aberrant formation of neutrophil extracellular traps (NETs) by neutrophils may represent a novel mechanism by which they contribute to the pathogenesis of arthritis (Song et al., 2020; Chamardani & Amiritavassoli 2022). NETs are fibrous structures comprising a decondensed chromatin meshwork decorated with neutrophil granules and cytoplasmic proteins generated from a specialized neutrophil death (NETosis). Through several mechanisms, NETs in RA enhance the cartilaginous component’s immunogenicity, thereby damaging the articular cartilage. Firstly, potent enzymes are released into the extracellular space during NETs formation resulting in tissue injury (Carmona-Rivera et al., 2020). Secondly, NETs cause citrullinated autoantigens and immunostimulatory molecules to externalize, leading to the upregulated expression of pathogenesis-related epitopes, contributing to RA-fibroblast-like synoviocyte (FLS) activation, which infiltrates the cartilage to trigger proinflammatory responses (Khandpur et al., 2013). More importantly, inflammatory cytokines such as IL-8 and IL-17 recruit neutrophils triggering the vicious cycle of NETs formation and autoantibody biogenesis (Song et al., 2020). Therefore, NETs might be taken as a novel potential target for RA therapy.

Deoxyribonuclease I (DNase) is a natural endonuclease responsible for the hydrolysis of extracellular deoxyribonucleic acid (DNA) and can prevent NETs formation (Meng et al., 2012). DNase has been used to treat several autoimmune diseases, including psoriasis, systemic lupus erythematosus, and antiphospholipid antibody syndrome (Kessenbrock et al., 2009; Lin et al., 2011; Nakazawa et al., 2012; Sangaletti et al., 2012; Katkar et al., 2016). Enhanced NETs generation in the serum and synovial fluid was observed in patients with RA, suggesting that impaired DNase activity might contribute to NETs persistence (Khandpur et al., 2013; Corsiero et al., 2016). Some experiments have reported that disruption of the NETs architecture caused by DNase significantly decreased their stimulatory effect on FLSs (Khandpur et al., 2013). Moreover, polydatin-mediated inhibition of NETs formation could prevent collagen-induced arthritis (CIA) in mice (Yang et al., 2019). Consequently, exogenous administration of agents such as DNase to inhibit or disrupt the formation of NETs may be used for managing RA.

**Scheme 1.**
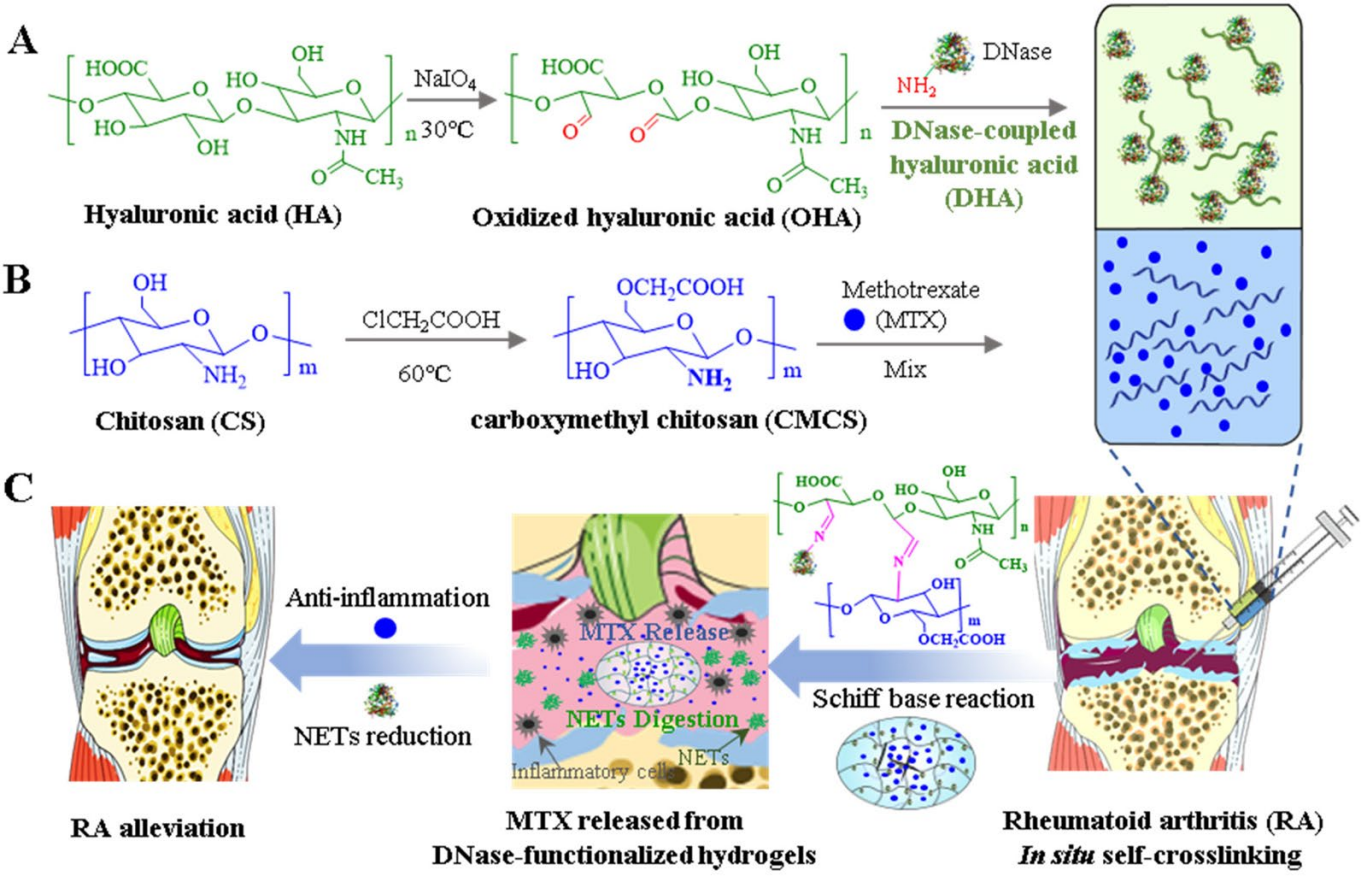
An illustration of DNase-functionalized hydrogel with MTX loading for rheumatoid arthritis treatment. (A) The synthesis of DNase-coupled oxidized hyaluronic acid. (B) The prepared process of carboxymethyl chitosan. (C) Intra-articular injection of DNase-functionalized hydrogel for the treatment of RA.

DNase has low toxicity and has been applied in murine models of breast cancer, in patients with lupus, or patients with lung damage due to cystic fibrosis (Cantin 1998; Masucci et al., 2020). However, protein therapeutics, including DNase, have a short half-life and must be repeatedly administered to patients to achieve a curative benefit (Zaman et al., 2019). Optimized drug delivery and controlled release systems might combat the limited half-life of DNase in RA treatment. Hydrogel is characterized by tenable properties, degradation that can be controlled, and can protect labile drugs that can be broadly investigated as local drug delivery systems, particularly in RA therapy (Liu & Scherman 2018; Tan et al., 2018; Chen XM et al., 2022). Herein, DNase was coupled with oxidized hyaluronic acid (OHA) by a Schiff base reaction to extend the half-life for efficient NETs disruption. Hydrogels have been reported with preeminent adhesive ability (Xue et al., 2021; Zhang et al., 2022), which is in favor of DHA retention at the articular cavity. Accordingly, carboxymethyl chitosan (CMCS) was crosslinked with DNase-modified OHA to form an injectable, degradable, and biocompatible hydrogel (DHY) to further strengthen the adhesive ability at the articular cavity. The chemical modification did not influence DNase activity. Importantly, the DNase hydrogel could digest the NETs structure and decrease inflammatory cytokine expression *in vivo*. Methotrexate (MTX) was taken as a therapeutic drug and encapsulated into the system exhibiting a synergistically ameliorative effect on mice with RA (Scheme 1). In conclusion, our findings demonstrate the potential application of DNase in RA treatment and the development of an injectable hydrogel system to extend the half-life of DNase.

## Material and methods

### Materials

DNase, phorbol 12-myristate 13-acetate (PMA), and high molecular weight sodium hyaluronate were procured from Sigma-Aldrich. Chitosan (deacetylation degree of 95%) and sulfo-cyanine5.5 (Cy5.5) was procured from Energy Chemical (Shanghai, China). Sodium periodate (NaIO_4_) was procured from KeLong Chemical (Chengdu, China). Anti-neutrophil elastase was procured from Abcam Co., Ltd. 4′,6-diamidino-2-phenylindole (DAPI), Bicinchoninic acid (BCA) kit, 3-[4,5-dimethylthiazol-2-yl]-2,5 diphenyl tetrazolium bromide (MTT), and nitric oxide (NO) probe were procured from Beyotime Biotechnology (Nantong, China). PE-labelled anti-CD86 antibodies and FITC-labelled CD80 antibodies were obtained from BioLegend. RAW264.7 cells were procured from the Shanghai Cell Bank (Shanghai, China) and were cultured in Dulbecco’s modified eagle’s medium with high glucose, supplemented with 10% heat-inactivated fetal bovine serum, 2 mM glutamine, nonessential amino acids, and sodium pyruvate. The medium and supplements were procured from Thermo Fisher Scientific. Cells were cultured at 37 °C in a humidified chamber with 5% carbon dioxide. The solvents used in the studies were analytically pure and used directly without treatment.

### Synthesis and characterization of the hydrogel polymers

DNase-coupled hyaluronic acid (DHA) was prepared by connecting DNase to OHA according to previous reports (Su et al., 2010). Firstly, hyaluronic acid (HA, 1.0 g) was dissolved by stirring it in distilled water (250 mL) at room temperature. Secondly, 20 mL of an aqueous solution comprising NaIO_4_ (1.4 g) was added dropwise to the prepared HA solution under magnetic stirring and was maintained under dark conditions for 48 h. Ethylene glycol (1 mL) was used to stop the reaction. OHA was purified and collected by dialyzing and freeze-drying, respectively. For DHA preparation, DNase (1 mg/mL) was added dropwise to the oxidized OHA solution (2 mg/mL) with magnetic stirring under dark conditions at room temperature for 24 h. The reacted products were then dialyzed (molecular weight cut-off = 300 kDa) in distilled water for 24 h, and the purified DHA polymers were finally freeze-dried for the subsequent studies. CMCS was prepared according to a previous report (Bao et al., 2014). Chitosan (1 g) was dispersed in 75 mL of isopropanol under magnetic stirring at room temperature for 30 min before 20 mL of 10 N sodium hydroxide was added. The solution was magnetically stirred for 30 min, and 69 g of chloroacetic acid (dissolved in 20 mL of deionized water) was added dropwise. The reaction was continued at 60 °C for 3 h, following which 200 mL of methanol was added. The solid product was then filtered, suspended in 80% of methanol, and extensively washed with 80% ethanol, following which it was filtered for 2 h. The product was dissolved in deionized water and freeze-dried. The polymers were characterized with ^1^H NMR spectra (Bruker ECX 400, Germany).

### Preparation and characterization of DNase-functionalised hydrogel (DHY)

DHY was prepared by directly mixing DHA and CMCS. In brief, different mass ratios of DHA (100 mg/mL) and CMCS (60 mg/mL) were blended at 37 °C. The inverted test tube method was used to study the gelling ability. The morphology of DHY was examined using a scanning electron microscope (SEM). The crosslinked reaction (Schiff base) was confirmed using Fourier-transform infrared spectroscopy (FT-IR).

### *In vitro* cell viability assay for DHY

Cell viability mediated by the different treatments was evaluated using the 3-(4,5-dimethylthiazol-2-yl)-2,5-diphenyltetrazolium bromide (MTT) assay. RAW264.7 were seeded on a 96-well plate with a density of 5 × 10^3^ cells per well for CMCS and DHA investigations. After 12 h, the cells were added to different concentrations of CMCS or DHA and were cultured for 48 h. Following this, 10 μL of MTT (5.0 mg/mL, Sigma Aldrich) was added to each well and was further incubated for 4 h. The purple crystals were dissolved by 100 μL of dimethyl sulfoxide. The absorbance was measured at 570 nm in a microplate reader (TECAN, F50). RAW264.7 cells were seeded in the bottom chamber for 12 h for the cytotoxicity assay of DHY. Following this, different concentrations of DHY were divided into small pieces and incubated with RAW264.7 cells in the upper chamber. The cells were further cultured for 48 h, following which MTT assay was performed as mentioned above.

### *In vitro* study of drug release

CMCS solution (60 mg/mL) comprising MTX (10.0 weight% of the total polymers) was mixed with 100 mg/mL of the DHA solution for preparing MTX-loaded DHY. The free drug and ethanol were removed by rinsing and centrifugation. MTX-contained DHY was separately suspended in phosphate buffer (PB, pH 7.4) and acetate buffer (pH 6.5) by slow shaking at 37 °C. The release medium was freeze-dried, and the amount of MTX was determined by high-performance liquid chromatography according to a standard curve (Ultramate 3000, Thermo Fisher, USA) with ultraviolet detection at 304 nm. Release experiments were conducted in triplicate, and the results are presented as the average ± standard deviation.

### DNA-cleavable function assay

A double-stranded DNA (plasmid DNA) was used as a substrate to evaluate the cleavability of DHA, according to a previous report (Kovaliov et al., 2018). In brief, 4 µg of plasmid DNA was added to PB containing Ca^2+^ and Mg^2+^ (6.25 mM) with a final volume of 40 µL. The reaction mixture was incubated at room temperature for 5 min, following which 8 µL of free DNase or 1 mg/mL of DHA was added. The reaction was incubated at 37 °C for 30 min. After the incubation, 1% agarose gel electrophoresis was used to analyze 20 µL of the reactive mixture.

### NETs structure digested by DHA

Neutrophils were isolated from bone marrow as previously described (Swamydas & Lionakis 2013). All animal experiments were carried out in compliance with the Animal Management Rules (Ministry of Health, People’s Republic of China) and the guidance for the Care and Use of Laboratory Animals (The Animal Ethics Committee of the First Hospital of Jilin University). The mice were euthanised and immersed in 70% ethanol. The bone marrow from the femur and tibia was flushed using a sterile syringe filled with iced phosphate-buffered saline (PBS). A 70 µm cell strainer was used to collect the bone marrow into a 50 mL conical tube. Red blood cell lysis buffer was added to the cell suspension. After lysing the red blood cells, the cells were centrifuged for 7 min at 1,400 rpm at 4 °C, following which they were rinsed with PBS. Neutrophils were separated from the bone marrow cells using the density gradient centrifugation protocols. The separated neutrophils were cultured in confocal dishes and stimulated with PMA (100 nM) for 4 h at 37 °C to promote NETs formation (Yuen et al., 2016). Free DNase and DHA were incubated with the PMA-stimulated neutrophils for 30 min. The cells were fixed in 4% paraformaldehyde for 10 min and permeabilised with Triton × 100 for a maximum of 5 min. Tris-buffered saline with 0.1% Tween® 20 detergent was used to rinse the cells, following which they were blocked with 5% bovine serum albumin and stained with rabbit polyclonal anti-elastase antibody (1:50 dilution, Abcam) and AlexaFluor 488-conjugated goat anti-rabbit immunoglobulin G secondary antibodies. The DNA was stained with DAPI and was observed under a confocal microscope (Zeiss, Germany).

### Flow cytometry analysis

M0 macrophages were cultured in the lower layer of the transwell system. Neutrophils separated from CIA mice were stimulated with 100 nM of PMA for 2 h. These neutrophils were then transferred to the upper layer of the transwell system and were incubated with or without precut DHY for 48 h. Flow cytometry analysis was used to investigate RAW264.7 cell polarization. PE-labelled anti-CD86 antibodies were used to stain the M1 macrophage marker. RAW264.7 cells incubated with lipopolysaccharide + interferon-γ were considered as the positive control. The macrophages were incubated with CD86 antibodies at 4 °C for 30 min under dark conditions. The expression of CD86 was determined by flow cytometry (BECKMAN CytoFLEX) and analyzed using FlowJo software after washing the macrophages thrice.

### Cellular NO release detection

Fluorescence probe (DAF-FM DA, Beyotime) was utilized to detect the effect of DHY on NETs-induced NO generation. Briefly, RAW264.7 was cultured in the lower layer of transwell system. Neutrophils separated from mice with CIA were stimulated with 100 nM of PMA for 2 h. These neutrophils were then transferred to the upper layer of the transwell system and were incubated with or without precut DHY for 24 h. After that, DAF-FM DA was added to the culture medium of RAW264.7 and incubated for 30 min. The cells were stained with DAPI and then examined by fluorescence microscope or flow cytometry.

### Enzyme-linked immunosorbent assay

The levels of TNF-α and IL-6 were detected with commercial kits following the manufacturer’s instructions (Hangzhou Lianke Biotechnology Co., Ltd, Hangzhou, China). Briefly, RAW264.7 was cultured in the lower layer of transwell system. Neutrophils separated from mice with CIA were stimulated with 100 nM of PMA for 2 h. These neutrophils were then transferred to the upper layer of the transwell system and were incubated with or without precut DHY for 48 h. After that, 100 μL of culture medium from RAW264.7 was used to perform ELISA assay.

### Establishment of the CIA model

The CIA mouse model was established using collagen immunization according to previous reports (Brand et al., 2007; Kim HJ et al., 2015). Briefly, bovine type II collagen was dissolved in 0.1 M acetic acid at 4 °C (2 mg/mL) and then emulsified with an equal volume of complete Freund’s adjuvant (5 mg/mL, Sigma). The collagen solution (100 μL) was injected subcutaneously into 8-week-old male DBA/1J mice at the base of the tail (Hangzhou Ziyuan Experimental Animal Technology Co., Ltd., Hangzhou, China). On the 21^st^ day, the DBA/1J mice received a booster injection of bovine type II collagen emulsified in incomplete Freund’s adjuvant.

### *In vivo* DNase release behavior of different formulations

The CIA mice received an intra-articular injection of free Cy5.5 and DHY-encapsulated Cy5.5 (Cy5.5@DHY, Cy5.5: 20 μg), respectively. The fluorescence was monitored by an IVIS Spectrum system (IVIS®Lumina™, USA) (at an excitation of 673 nm) at different time intervals. For *in vivo* DNase release studies, Cy5.5-labelled DNase (Cy5.5-DNase), hydrogel-encapsulated Cy5.5-DNase, and Cy5.5-DNase modified hydrogel (DHY) were performed with intra-articular injection, respectively. The IVIS Spectrum system was used to analyze the *in vivo* biodistribution of fluorescence at different time intervals. The fluorescence was quantified by measuring the average radiant efficiency in the knee joint region using the Living Image software.

### *In vivo* therapeutic efficacy evaluation

After 24 days of immunization, the CIA mice were randomly divided into five groups (*n* = 5), followed by intra-articular injection of different formulations (10 μL): PBS, nonfunctional hydrogel (HY), HY-mediated encapsulation of DNase (DNase@HY), DNase-modified HY (DHY), and DHY-encapsulated MTX (DHY@MTX). The mice were injected only once. MTX was administered at a dose of 0.2 mg/kg. Paw inflammation was assessed every other day, starting on day 24, after arthritis induction. Paw inflammation was determined by examining the paw thickness using a vernier caliper and by the scoring standard as follows (Yin et al., 2020): 0, normal; 1, erythema and mild swelling restricted to one toe; 2, erythema and mild swelling in more than one toe, but not the entire paw; 3, erythema and moderate swelling extending to the entire paw; 4, erythema and severe swelling on the whole paw and ankle. The ankle joints were dissected, fixed in 4% paraformaldehyde for 48 h, and decalcified with 15% neutral ethylenediaminetetraacetic acid solution at 25 °C on the 42^nd^ day. Decalcified tissues were embedded in paraffin and sliced into 5 μm-thick sections. Hematoxylin–eosin (H&E) staining, immunohistochemistry (IHC), and safranin O/fast green staining were performed on these thin sections. For H&E staining assay, the blocks were sliced, deparaffinized, and rehydrated through gradient alcohol. Endogenous peroxidase activity was inhibited by incubating with 3% of H_2_O_2_. Antigen retrieval in these tissues was performed by microwave heating in 0.01 M of citrate buffer (pH 6.0). Polyclonal anti-mouse TNF-α and IL-6 antibodies (1:100) were used to detect the antigens. An EnVision peroxidase kit (Dako, Agilent Technologies, Inc., Santa Clara, CA, USA) was performed to detect primary antibodies. Then, the tissues were incubated in 3,3′-diamino benzidine (Dako, Glostrup, Denmark, Agilent Technologies, Inc.), re-stained with hematoxylin, dehydrated through gradient alcohol, cleared in xylene, and cover slipped with permanent mounting media. Safranin O/Fast Green staining Kit (G1053, Servicebio, Wuhan, China) was performed to stain the tissues. Briefly, deparaffinize and rehydrate the tissues to distilled water and then stain them with Fast-Green solution for 10 min. The excess stain solution was removed by washing to colorless. Then, rinsing the tissues in hydrochloric acid alcohol slight to differentiate, wash in tap water. Finally, stain them in Safranin O solution for 30 s, and dehydrate in absolute alcohol. Clear in xylene for 5 min and mount with permanent mounting media.

### Statistical analyses

Results are presented in mean ± standard error of the mean. Statistical tests were carried out using GraphPad Prism (GraphPad Software). The significance of differences between groups was estimated by two tailed Student’s t-test or one-way ANOVA. The level of significance was set at probabilities of *p < 0.05, **p < 0.01, and ***p < 0.001.

## Results

### Preparation and characterization of DHY

**Figure 1.**
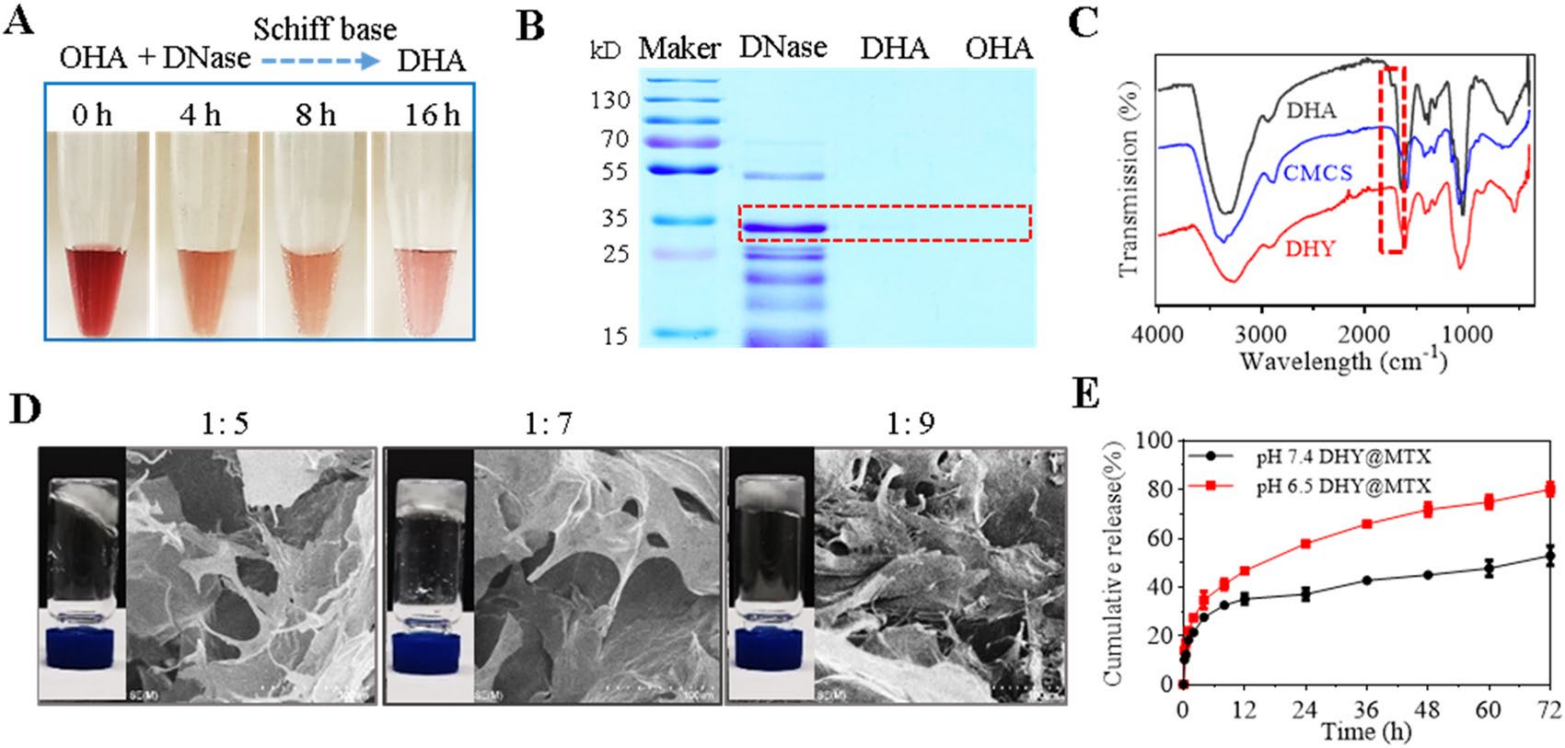
Characterization of DNase-functionalized hydrogel. (A) Color change for the indication of Schiff base reaction between OHA and DNase for different time. (B) Coomassie blue staining of free DNase, DNase-modified OHA (DHA) and free OHA. (C) FT-IR spectra of DHA, CMCS and DHY. (D) Tube inversion experiments and SEM images of DHY with different mass ratio of DHA and CMCS. (E) The cumulative release of MTX from DHY under different pH conditions.

**Figure 2.**
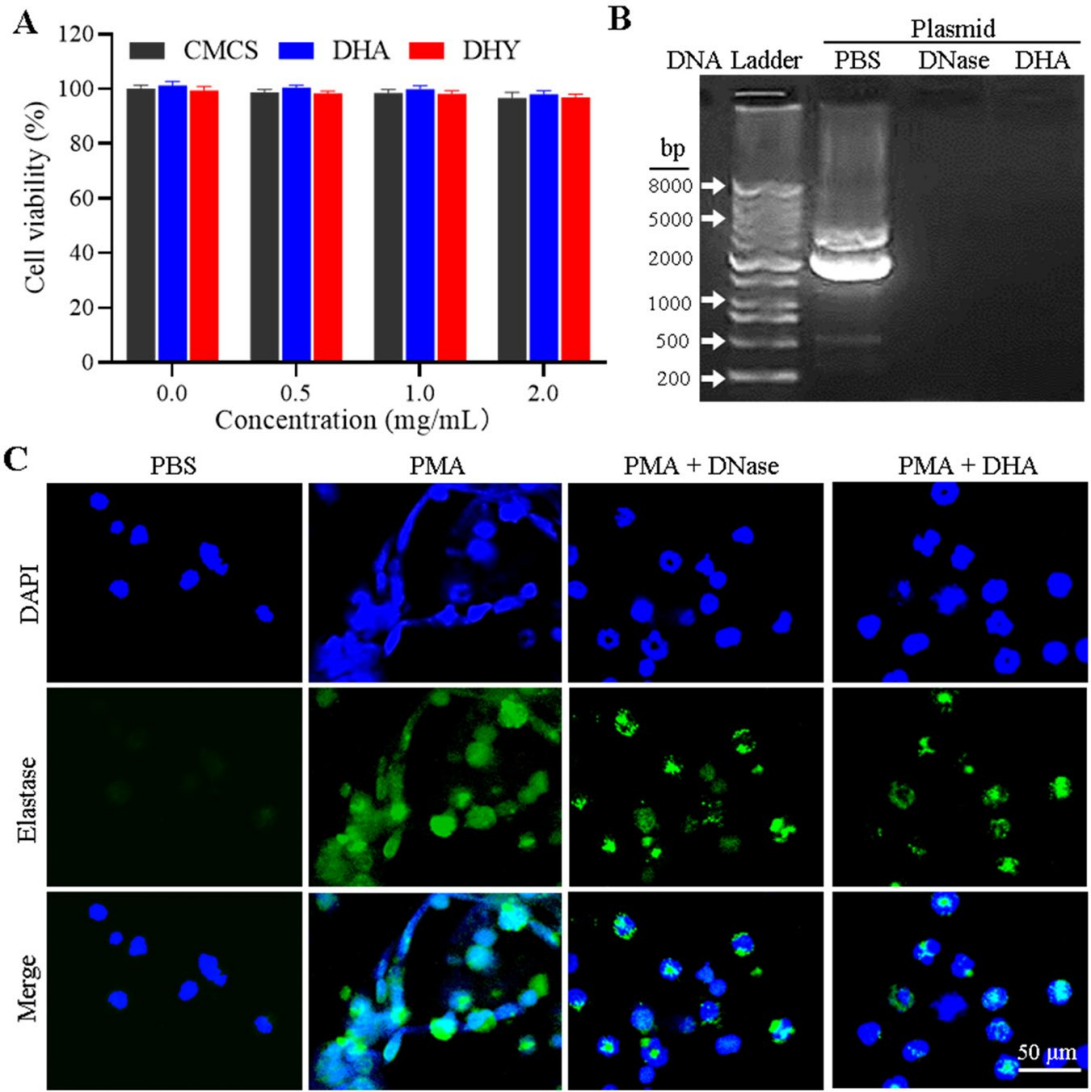
*In vitro* NETs digestion mediated by DHA. (A) Cytotoxicity of CMCS, DHA, and DHY on RAW264.7 cells determined by MTT assay. Cell viability measurements were performed after incubating the RAW264.7 cells with different concentrations of the formulations for 48 h. (B) Endonuclease activity of DHA. Plasmid DNA was incubated with 1 mg/mL of free DNase or DHA at 37 °C for 30 min. Control DNA was incubated in parallel without enzyme addition. The digestion product was run on 1% agarose gel. (C) Neutrophils were stimulated with PMA (100 nM) for 2 h followed by incubation with free DNase and DHA for 30 min. Neutrophils/NETs were washed and stained with 4′,6-diamidino-2-phenylindole and antibodies for elastase detection (green).

Based on physically or covalently crosslinking polymers like HA and chitosan, injected hydrogels have been widely used in protein drug delivery (Mantha et al., 2019). However, the therapeutic effect of DNase delivered by a hydrogel to target NETs structure digestion for RA management has not been evaluated. To address this issue, HA was successfully oxidized using NaIO_4_ (Figure S1) and covalently coupled with DNase via the Schiff base reaction between the amino groups from the enzyme and the aldehyde groups of OHA. The conjunction of DNase with OHA was confirmed by Purpald experiments, indicated by the color change following the reduction of the aldehyde group. As illustrated in Figure 1(A), the color gradually faded as the reaction time extended. This demonstrated that the aldehyde group reacted with DNase. Additionally, Coomassie blue staining was used to confirm the coupling reaction because the high molecular weight of DHA prevented it from moving in gel electrophoresis (Figure 2B and S2). The mass ratio for DNase in OHA was 15.5% determined by BCA assay. Chemical modification of OHA with DNase would facilitate sustainable degradation of the NETs structure. Furthermore, the residual aldehyde group on DHA could rapidly crosslink with CMCS (Figure S3) to form DHY. The Schiff base reaction between the amino groups of CMCS and the aldehyde groups of DHA contributed to DHY gelation, which was confirmed by FT-IR. The aldehyde group disappearance from DHY on FT-IR demonstrated that DHA and CMCS were successfully crosslinked (Figure 1C). Different mass ratios of DHA and CMCS were mixed, and the hydrogel stability and gelling time were recorded to study the mass ratios of DHA and CMCS in terms of the gelling properties. The tube inversion experiments and SEM images demonstrated that DHA and CMCS could form stable hydrogels in 30 s when the ratio was 1:7 for DHA and CMCS, respectively, which was used in the subsequent studies (Figure 1D). The swelling experiments also exhibited parallel results in Figure S4. Additionally, hydrogels have been widely used for the localized controlled release of therapeutics. Therefore, MTX, a commonly used drug for treating RA, was encapsulated into DHY and contributed to the hydrophobic interaction, wherein the loading efficiency could reach up to 78.7%. The release behaviors were studied under different pH conditions since the articular cavity in RA was characterized by a weak acid environment (Goldie & Nachemson 1969). More than 30% of MTX was released at a pH of 7.4 and 6.5 during the first 8 h, which might be attributed to the sudden release and fast diffusion at the beginning of DHY swelling (Figure 1E). However, a pH of 6.5 significantly accelerated MTX release, wherein approximately 80% and 50% of the MTX were released from DHY at the pH of 6.5 and 7.4, respectively. These results indicate that an acid environment might promote the degradation and dissociation of DHY.

**Figure 3.**
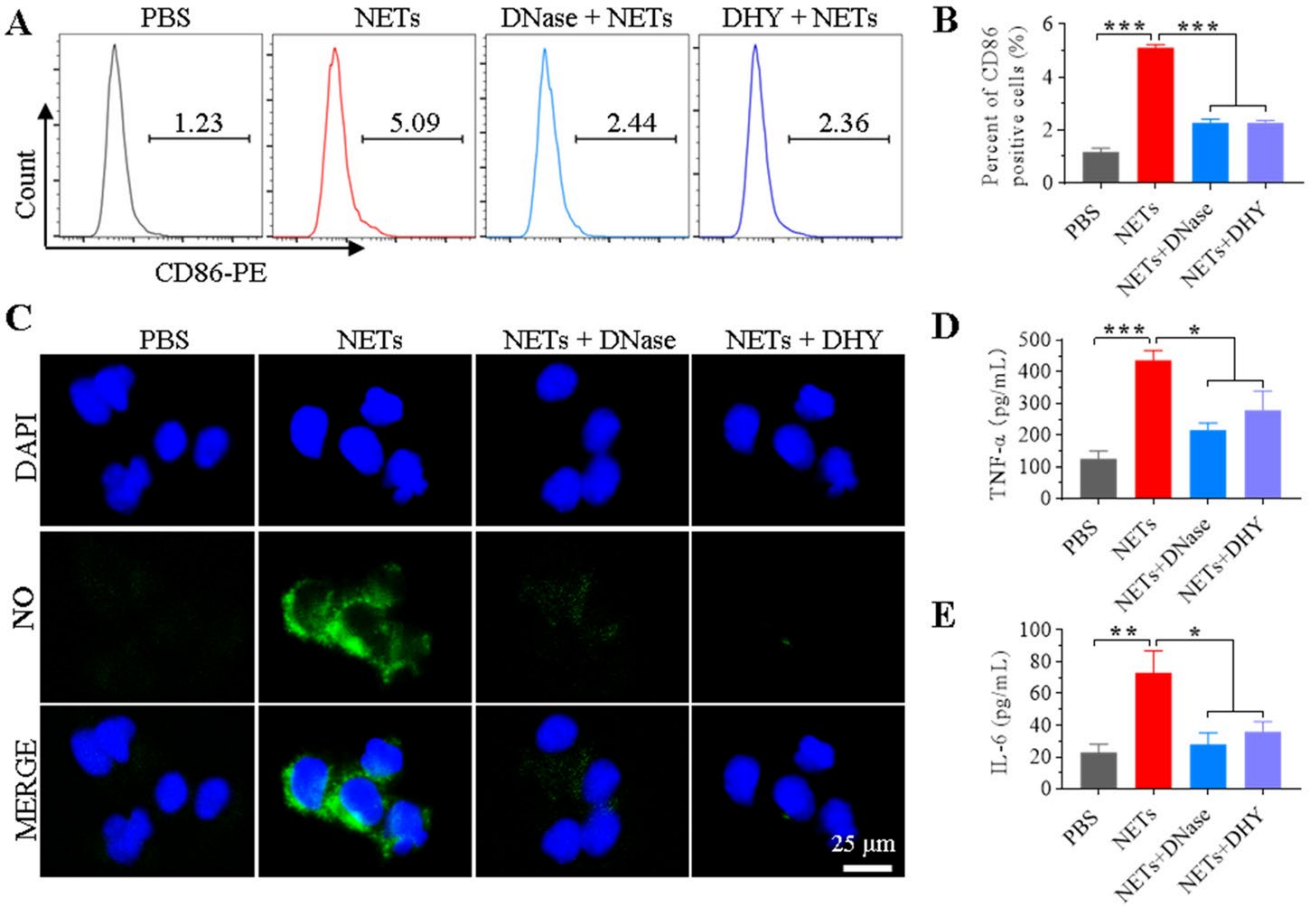
DNase-functionalised hydrogel inhibited NETs-promoted M1 macrophage polarization. Neutrophils separated from mice with collagen-induced arthritis were stimulated with PMA (100 nM) for 2 h, followed by incubation with RAW264.7 cells in the transwell system. (A-B) RAW264.7 cells were cultured in the lower chamber and incubated with NETs upon different treatments (upper chamber) for 48 h. The CD86 of M1 macrophage marker was determined by flow cytometry. (C) Fluorescence detection for nitric oxide in RAW264.7 cells with different treatments. (D-E) Enzyme-linked immunosorbent assay for inflammatory factors in the RAW264.7 cell supernatants.

**Figure 4.**
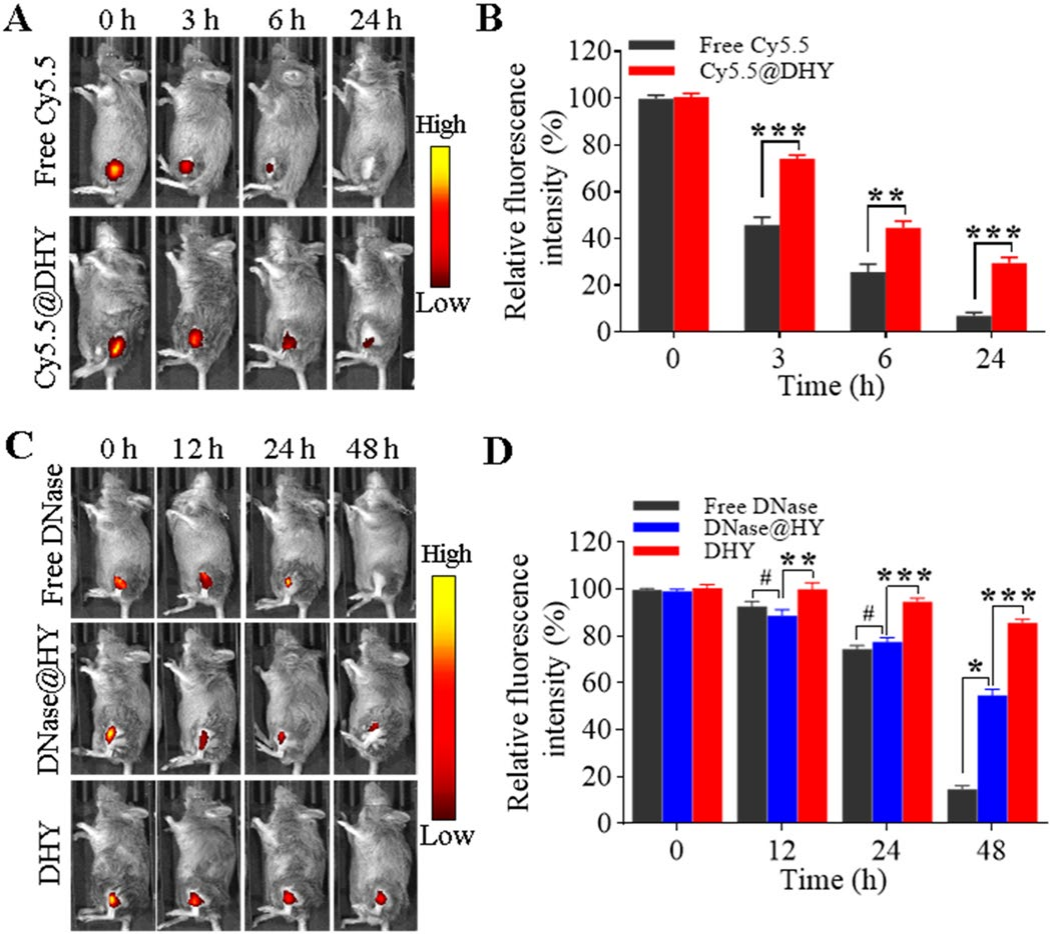
In vivo controlled release behaviors of DHY. (A-B) IVIS images during different time intervals after the intra-articular injection of free Cy5.5 or DHY-encapsulated Cy5.5 (A) and determination of the fluorescence intensity (B). (C-D) IVIS images for different periods after the intra-articular injection of Cy5.5-labelled DNase with different formulations in CIA mice (C) and relative fluorescence intensity determined at each time interval (D).

### *In vitro* NETs digestion mediated by DHA

The biocompatibility of different components in DHY were evaluated on a murine macrophage-like cell line (RAW264.7) to investigate the potential clinical applications. Different concentrations of CMCS, DHA, and DHY were incubated with RAW264.7 cells for 48 h (Figure 2A). Neither of the polymers exhibited obvious cell viability reduction at the highest concentrations, indicating that DHY had good biocompatibility. Importantly, DHA could digest plasmid DNA, indicating that the chemical modification did not influence DNase enzyme activity (Figure 2B). Accordingly, whether DHA could digest the NETs structure was studied by confocal assay. Neutrophils were collected from mouse bone marrow and treated with PMA, followed by free DNase or DHA treatment. The typical NETs structure was observed when the neutrophils were incubated with PMA for 2 h, as shown in Figure 2(C). However, the NETs were disrupted and disappeared after DHA or free DNase treatment. These results demonstrated that DNase-modified polymers did not alter enzyme activity and could eliminate the NETs structure, which might be advantageous to the *in vivo* digestion of NETs. Furthermore, the effect of DNase, DHA and DHY on the activation and cell viability of neutrophils after treatment of PMA was investigated. As shown in Figure S5A, PMA treatments could significantly increase the release of reactive oxygen species (ROS), while DNase, DHA or DHY did not change the level of ROS released from the neutrophils. Accordingly, DNase, DHA and DHY treatments showed no influence on the cell viability of PMA-treated neutrophils (Figure S5B).

**Figure 5.**
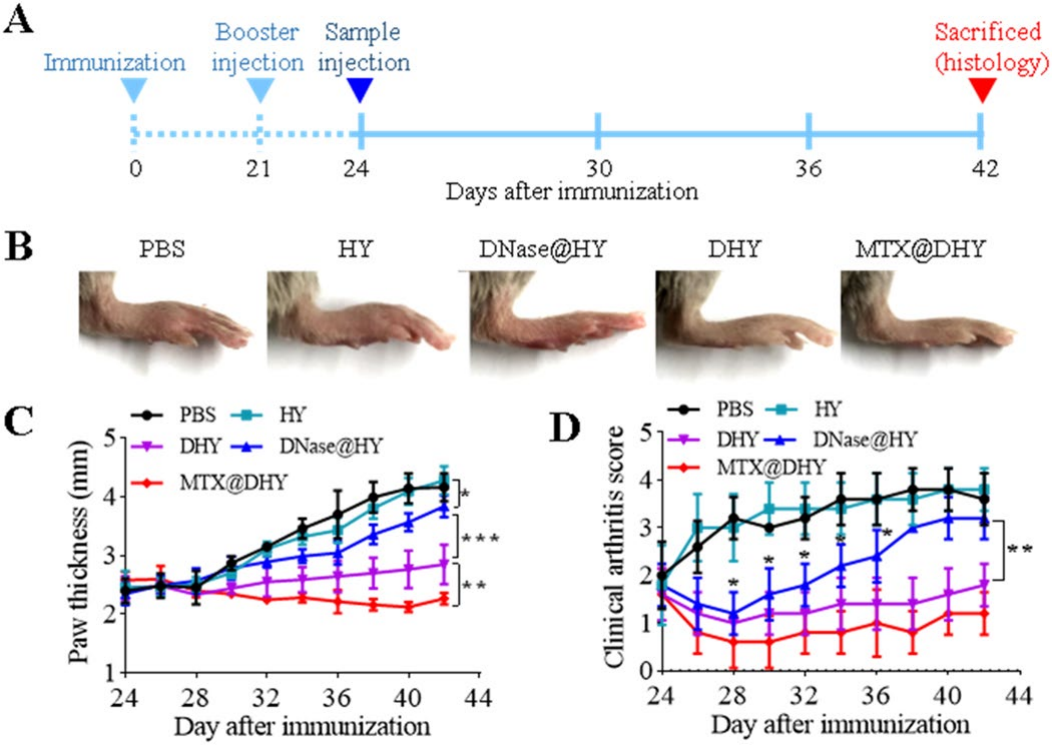
*In vivo* therapeutic effect of DHY in a collagen-induced arthritis mouse model. (A) Overall experimental timeline for in vivo therapeutic effect evaluation. (B) Representative images of arthritic paws from different groups at the end of the experiments. (C) Hind paw thickness and (D) arthritis scores.

### *In vitro* anti-inflammatory activity of DHY

NETs have been reported to increase the inflammatory response by activating macrophages for cytokine release (Hu et al., 2019; Josefs et al., 2020). Accordingly, the *in vitro* anti-inflammatory activity of DHA was examined on RAW264.7 cells. As shown in Figure 3(A), B, and S6–S8, NETs treatments could promote macrophage polarization from M0 to M1, indicated by increased CD86 and CD80 expression. However, free DNase or DHY treatment could significantly block M1 macrophage polarization induced by NETs incubation. The findings suggest that DHY might be crucial in reducing inflammation of RA. Nitric oxide (NO) serving as one of the important indicators released from inflammatory reaction could also stimulate the formation of NETs (Patel et al., 2010). Therefore, NO and inflammatory factors released from RAW264.7 cells were examined. Figure 3(C) and S9 show that the fluorescence intensity of the NO probe markedly increased after NETs treatment, demonstrating that NETs may mediate NO release and promote inflammation, both of which could be reversed by free DNase or DHY. Accordingly, the inflammatory cytokines of TNF-α and IL-6 in the RAW264.7 cell culture supernatant exhibited that DHY treatments could significantly decrease the secretion of inflammatory factors from NETs-incubated macrophages (Figure 3D and E).

### *In vivo* release behaviors of DHY

Injectable hydrogels have been widely used for drug delivery in RA management because of their controlled release character (Kim T et al., 2021). Therefore, to study the *in vivo* controlled release behavior of DHY, the fluorescence dye of Cy5.5 was used as a modal dye. The fluorescence of free Cy5.5 rapidly decreased and disappeared 24 h after the intra-articular injection, as shown in Figure 4(A) and B. In contrast, Cy5.5 encapsulated into DHY exhibited more durable fluorescence and maintained approximately 30% of the fluorescence. The *in vivo* release was quicker than that observed in the *in vitro* MTX release experiments, which might be explained by the fact that the modal dye of Cy5.5 is water soluble and easily diffused from DHY. Additionally, different formulations of Cy5.5-modified DNase were injected into the articular cavity of mice with CIA to study the behaviors of DNase *in vivo.* Strong fluorescence signals were found in the joint cavity of the mice following injections of free DNase, DNase@HY, and DHY (Figure 4C and D). Almost no fluorescence was detected for free DNase 48 h after injection. Moreover, the fluorescence intensity decreased by more than 40% when DNase was encapsulated into a hydrogel. However, only 5% of DHY’s fluorescence intensity decreased after 48 h, indicating that DNase was immobilized in the joint cavity and could be retained there for a long period. Moreover, long period monitoring of fluorescence intensity about DHY was performed indicating that about 6% of the fluorescence could be detected after 15 days of intra-articular injection (Figure S10). These results demonstrated that DHY had good *in vivo* stability and might exhibit long-acting functions to prevent the formation of NETs in the joint cavity and alleviate joint inflammation.

### *In vivo* therapeutic efficacy of DHY

CIA mice characterized with immunological and pathological features of human RA have been widely used as a model to investigate therapeutic agents for RA (Marty et al., 2001). Therefore, the combined therapeutic efficacy of DHY and MTX was evaluated on CIA mice (Figure 5A). One intra-articular injection of PBS, HY, DNase@HY, DHY, and MTX@DHY was administered in CIA mice with edema, erythema, and leg stiffness. Severe edema and erythema persisted in mice treated with PBS or HY in all the experimental periods. However, the pathological symptoms of mice treated with DNase@HY were alleviated and were almost relieved in mice treated with DHY and MTX@DHY (Figure 5B). The paw perimeter and arthritis scores (AI) were recorded every two days starting on day 24 when CIA was established. No significant difference in the paw perimeter and AI were reported among PBS, HY, and DNase@HY treatments at the end of experiments, which might be explained by the fact that DNase directly encapsulated in HY was easily released and degraded than that covalently coupled to polymers (Figure 4C and D). Accordingly, the mice injected with DHY exhibited a significant decrease in the paw perimeter and AI. Mice treated with a combination of DHY and MTX reached a value close to that of normal mice at the end of treatments. These results demonstrated that targeting NETs digestion could alleviate pathological symptoms of RA, which could be significantly strengthened by the combined application of MTX.

### Histological analysis of the knee joint

**Figure 6.**
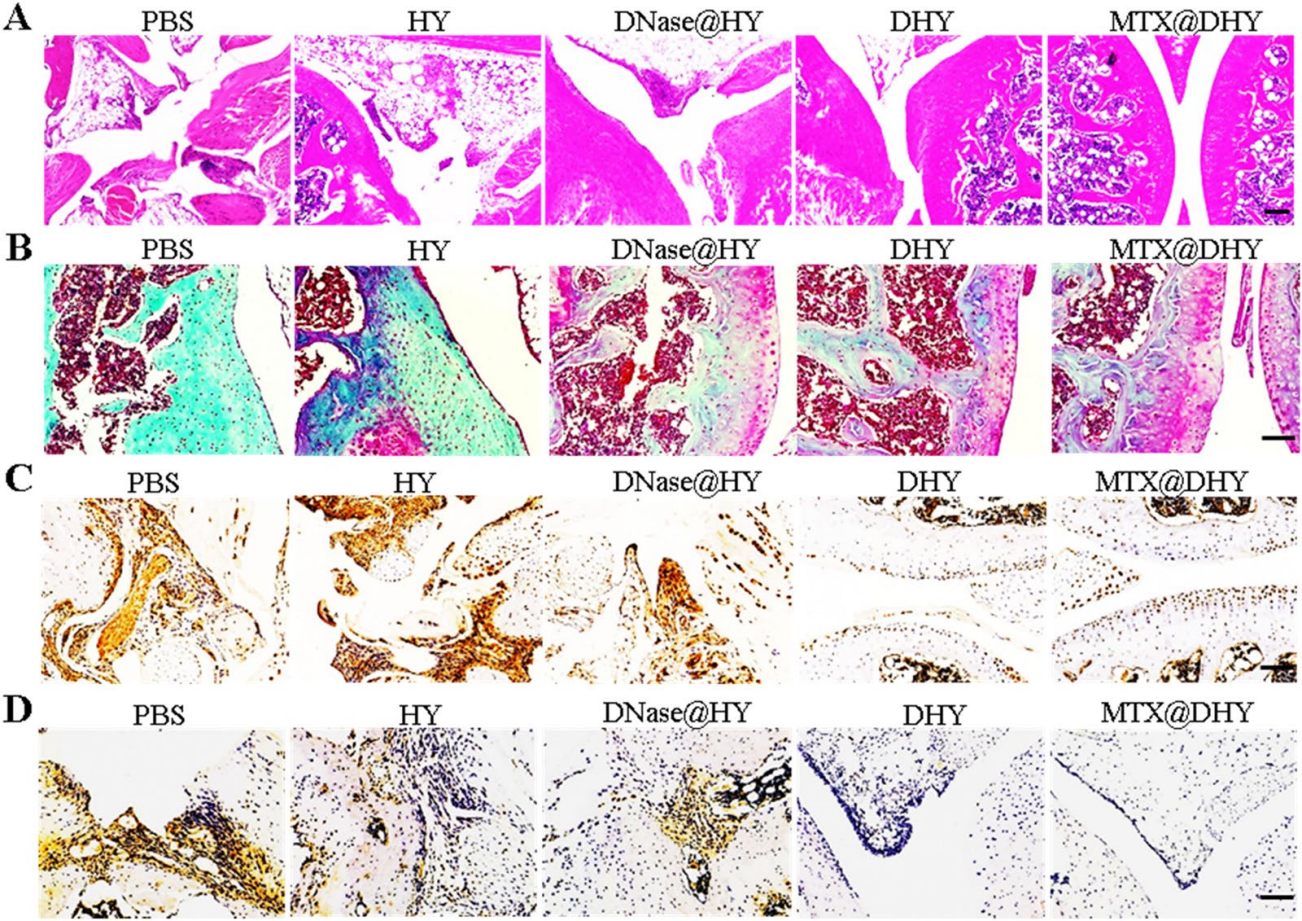
The combined anti-inflammatory effects of DHY and MTX on the RA model. (A-B) H&E (A) and safranin O/fast green (B) staining of the knee joint tissue. (C-D) IHC staining of tumor necrosis factor-α (C) and interleukin-6 (D). Scale bar, 100 μm.

The therapeutic effect of DHY was further evaluated by H&E and safranin O/fast green staining of the joint tissues. As shown in Figure 6(A), Severe cartilage destruction and erosion were observed in RA mice treated with PBS or HY. However, RA mice treated with DNase@HY presented with weaker pathological conditions compared with mice belonging to the PBS or HY groups. Mice belonging to the DHY group exhibited markedly reduced levels of synovial tissue hyperplasia and cartilage destruction. Furthermore, the combination of MTX and DHY exhibited the strongest therapeutic effect, indicated by the clear articular surface. These results were confirmed by safranin O/fast green staining for articular knee joints (Figure 6B). RA is characterized by a high inflammatory mediator expression. Accordingly, immunohistochemical staining was used to examine the classical inflammatory factors. DHY treatment could significantly decrease the expression of TNF-α and IL-6, which could be further reduced by combining MTX (Figure 6C and D). These results demonstrated that DHY-mediated *in vivo* digestion of the NETs structure could alleviate RA by decreasing the expression of inflammatory factors and blocking inflammatory signaling pathway feedback.

**Figure 7.**
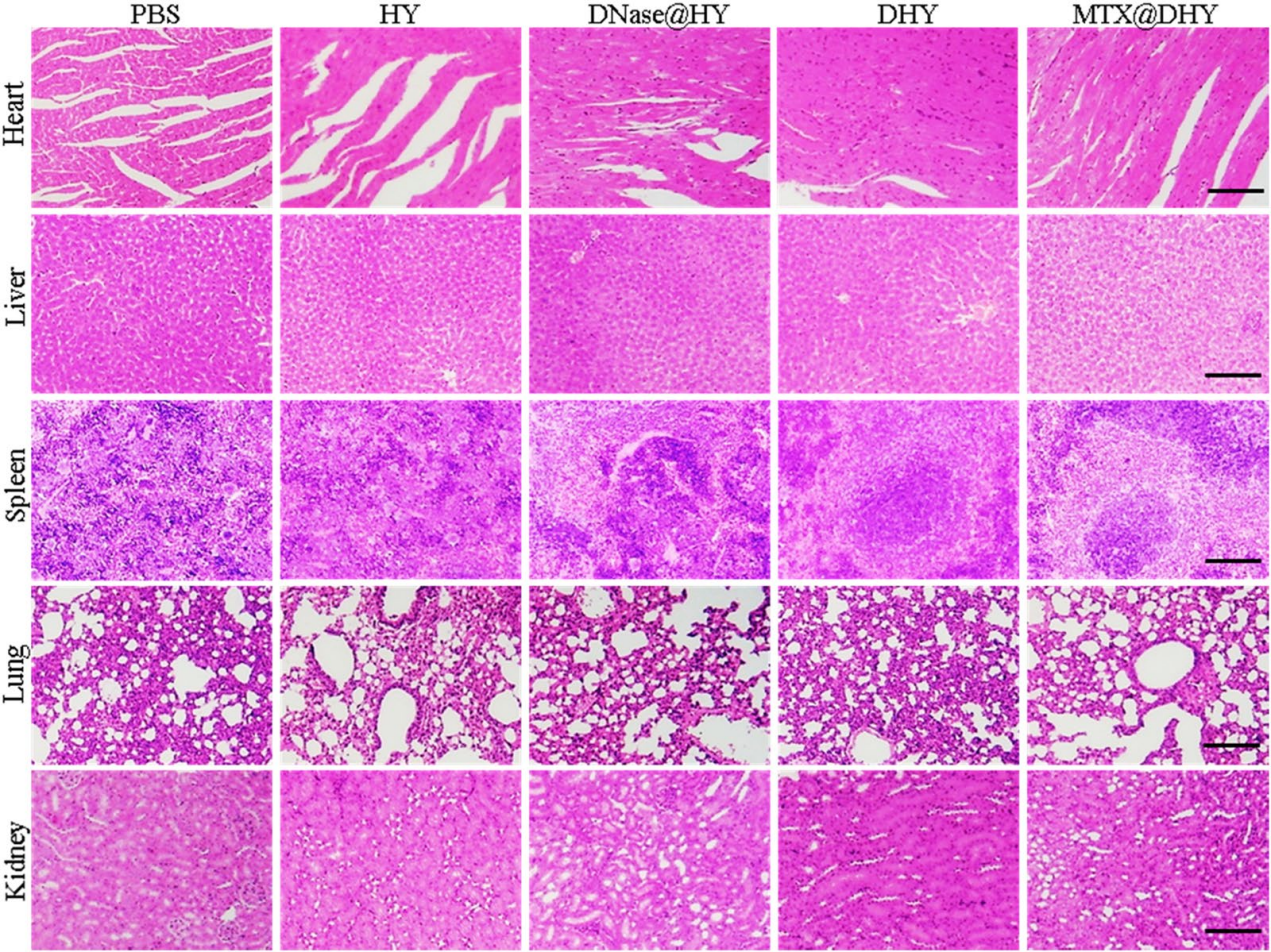
H&E staining of major organs tissues from different groups, scale bar, 200 μm.

The weight variations in the mice subjected to different treatments were monitored to investigate the biocompatibility of DHY. There were no significant differences in mouse weight among the mice subjected to different treatments during the experimental period (Figure S11). Furthermore, the biocompatibility of DHY was evaluated by performing H&E staining on the major organ tissues obtained from mice belonging to different groups. No pathological alterations were observed among the different organs, indicating that the intra-articular DHY injection did not cause systemic toxicity (Figure 7).

## Discussion

NETs have been reported to play an important role in the chronic autoimmune inflammatory diseases such as lupus, psoriasis, atherosclerosis and arthritis. Several mechanisms are involved in NETs-mediated aberrant inflammatory responses including activation of cGAS-STING, endosomal Toll-like receptors, NLRP3 inflammasome pathways, and small RNAs externalized in NETs for the induction of proinflammatory responses (Wigerblad & Kaplan 2022). In RA, neutrophils seem to have a central inflammatory role in initiating and perpetuating autoantigen exposure through NETs formation as these structures are a major source of autoantigens for the promotion of inflammatory responses (Chen W et al., 2018). Therefore, inhibiting neutrophil activation or NETs formation represent an attractive strategy for the treatment of RA. For instance, Yuan et al reported that the flavonoid quercetin could inhibit NET formation via suppressing ROS production and autophagy to serve as a therapeutic strategy in treating RA (Yuan et al., 2020).

Various groups have demonstrated that impaired NETs degradation resulting from the deficiency of DNase had an increased risk of autoimmune diseases (Wigerblad & Kaplan 2020). Accordingly, DNase, which has been approved by U.S. Food and Drug Administration for cystic fibrosis management, could be utilized to digest NETs to ameliorate the symptoms of RA. However, the low stability has been considered as the major limited factor for further application of DNase. Sun et al.’s study used a temperature-sensitive hydrogel to load DNase serving as control release system for epidural fibrosis therapy in a mouse model of laminectomy (Sun et al., 2022). In another work, DNase was successfully conjugated to microgels for more efficient digestion of NETs to treat thrombosis than free DNase (Hosseinnejad et al., 2021). Consequently, combination of DNase with optimized drug delivery system may play an important role in RA treatment.

In the present study, DNase was covalent connected to OHA based on Schiff base reaction. The chemical modification did not alter enzyme activity for NETs degradation. To date, hydrogels have been the most promising materials that are widely investigated as a potential therapeutic strategy in RA treatment. Here, carboxymethyl chitosan was used to crosslink with DNase-modified OHA to prepare injectable hydrogels. The DNase-conjugated hydrogels could be *in situ* formed after intra-articular injection of the polymers. In a collagen-induced arthritis model, DNase-functionalized hydrogels exhibited significant stronger activity on inflammation inhibition than hydrogel-mediated encapsulation of DNase. The results indicated that chemical connection of DNase to the hydrogels is a pivotal condition for the RA treatments. Previous studies demonstrated the pro-inflammatory role of NETs in the development of RA through activating innate immunity (Wigerblad & Kaplan 2022). Current strategies for RA treatment mainly focus on the inhibition of inflammatory signal pathway to decrease the secretion of inflammatory factors. Instead, our studies utilized the DNase-functionalized hydrogels to digest NETs to block inflammatory stimuli from the source. Although the current studies about DHY lacked detailed research on the mechanisms, the results supported the idea that NETs contribute to articular inflammation, and efficient drug delivery system loaded with DNase can be an alternative approach for the treatment of RA.

## Conclusions

NETs represent a novel mechanism that promotes the expression of inflammatory cytokines and chemokines that deteriorate the arthrosis microenvironment. Herein, DHY was successfully engineered for NETs digestion and applied to alleviate the RA. DNase was covalently coupled with OHA without altering the enzyme activity. Furthermore, DNase-modified polymers could rapidly crosslink with CMCS to form injectable hydrogels based on the Schiff base reaction, which could significantly increase the half-life of DNase in the articular cavity. The combination of DHY and MTX exhibited a prominent curative effect on a CIA mouse model and decreased the expression of inflammatory factors without obvious side effects. Consequently, the injectable hydrogel targeting NETs degradation represents a promising strategy for managing RA.

## Supplementary Material

Supplemental Material

## Data Availability

The raw data supporting the conclusions of this article will be made available by the authors without undue reservation.
