# Supplementary material for: An injectable hydrogel to disrupt neutrophil extracellular traps for treating rheumatoid arthritis
Source: Drug Deliv. 2023 Feb 1;30(1):2173332. doi: 10.1080/10717544.2023.2173332 (PMC9897762; doi:10.1080/10717544.2023.2173332)
Supplement: Supplemental Material [file IDRD_A_2173332_SM3813.docx]

Supplementary Material


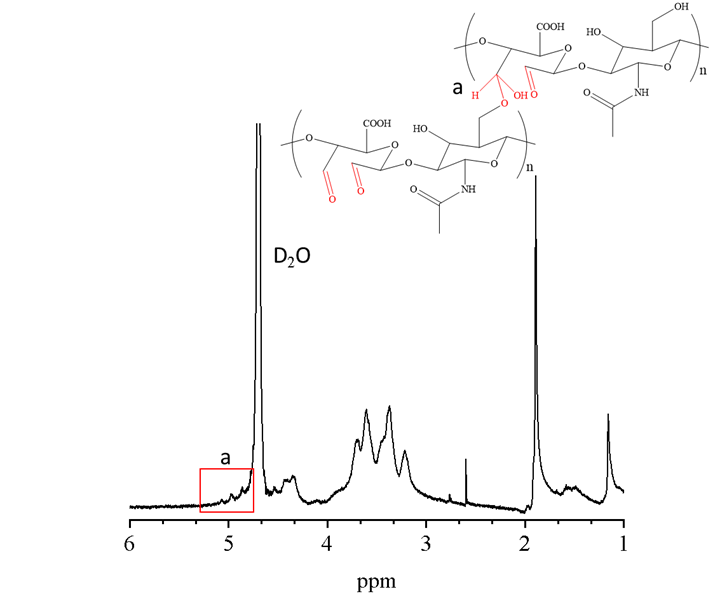


**Supplementary Figure 1.** ^1^H NMR spectra of biodegradable polymers of OHA.

**
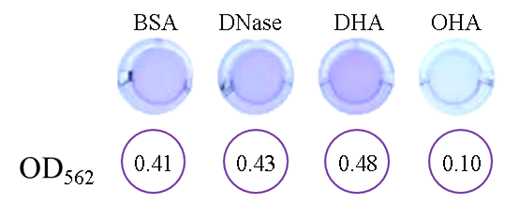
**

**S****upplementary Figure 2.** BCA assay for DHA


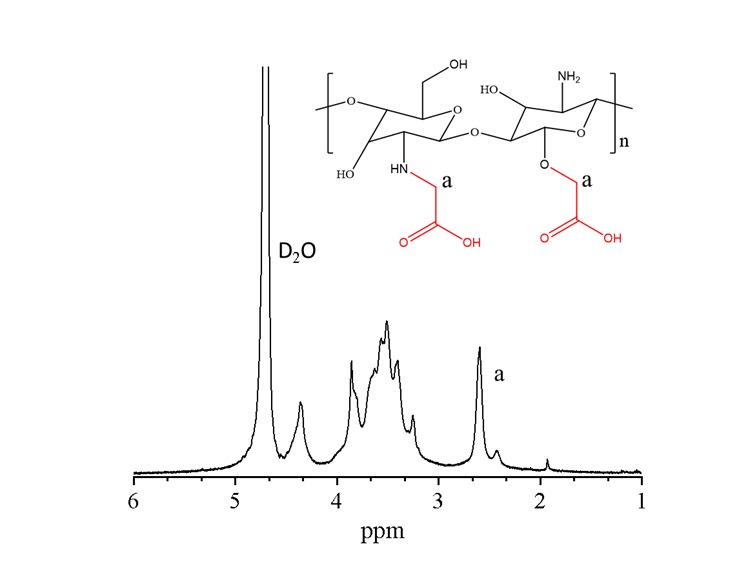

**Supplementary Figure 3.** ^1^H NMR spectra of biodegradable polymers of CMCS.

**Supplementary** **Figure 4.** The swelling ratios of hydrogels with different ratios of DHA and CMCS.


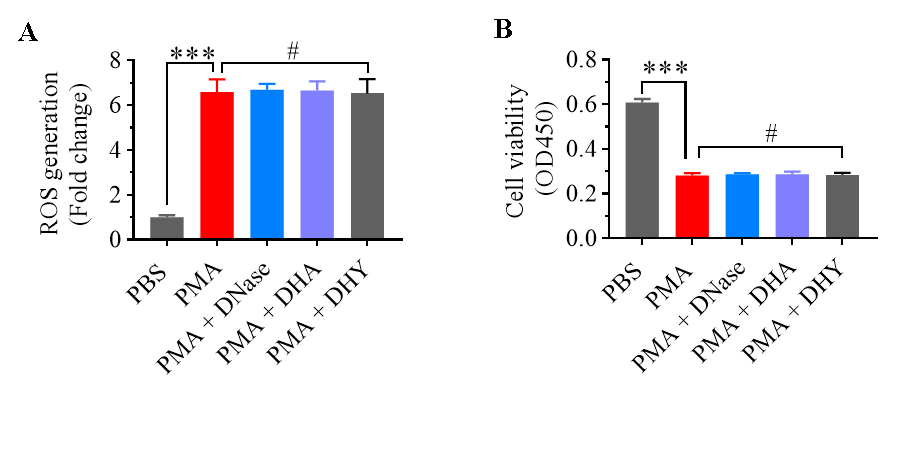


**Supplementary** **Figure 5.** (A) The freshly separated neutrophils were stimulated with PMA (100 nM) and incubated with different formulations of DNase for 4 h. The ROS release was quantified by flow cytometry using ROS probe of DCFH-DA. (B)The viability of neutrophils was tested by CCK-8. The freshly separated neutrophils were stimulated with PMA (100 nM) and incubated with different formulations of DNase for 4 h. The sign of # indicated no significant difference.


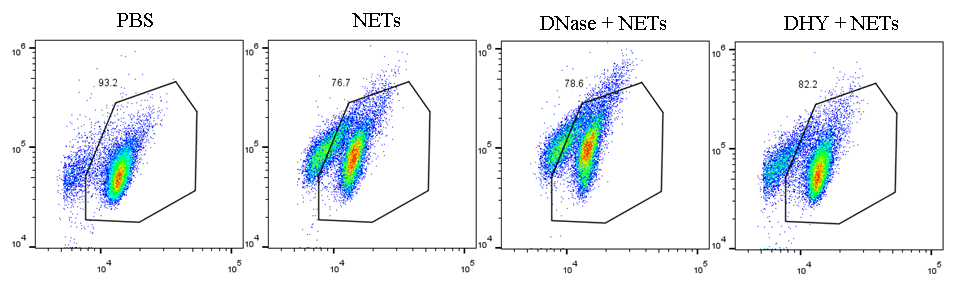
**Supplementary Figure 6.** Gating strategy for CD86 expression analysis.


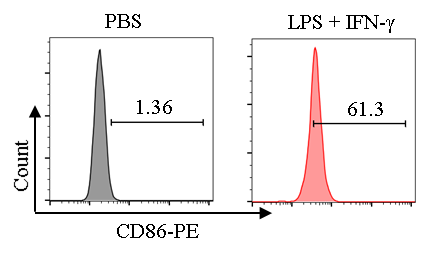


**Supplementary Figure 7.** Flow cytometry of RAW264.7 cells incubated with LPS (100 ng/mL) and IFN-γ（50 ng/mL) for 24 h.


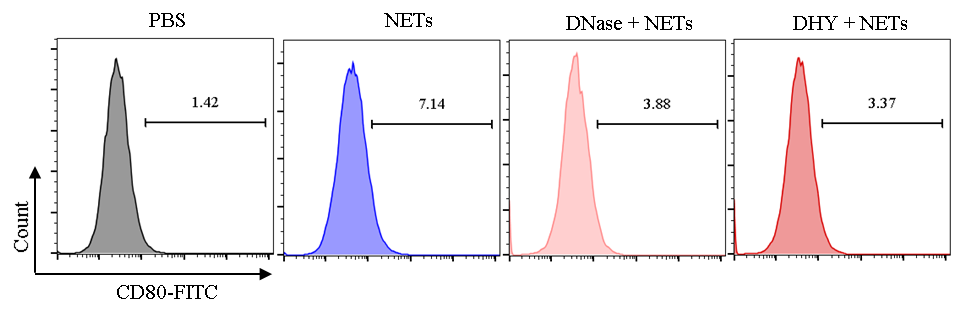


**Supplementary Figure 8.** Flow cytometry of CD80 expression on RAW264.7 cells incubated with NETs upon different treatments.


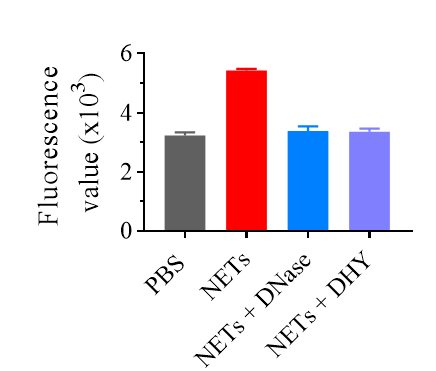


**Supplementary Figure 9.** Fluorescence value of NO probe determined by flow cytometry.


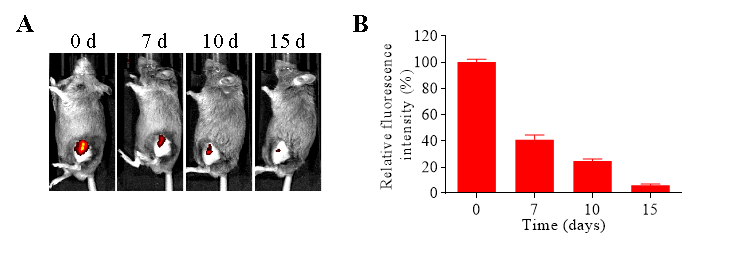


**Supplementary Figure 10.** IVIS images for different periods after the intra-articular injection of Cy5.5-labelled DNase -modified DHY in CIA mice (A) and relative fluorescence intensity determined at each time interval (B).


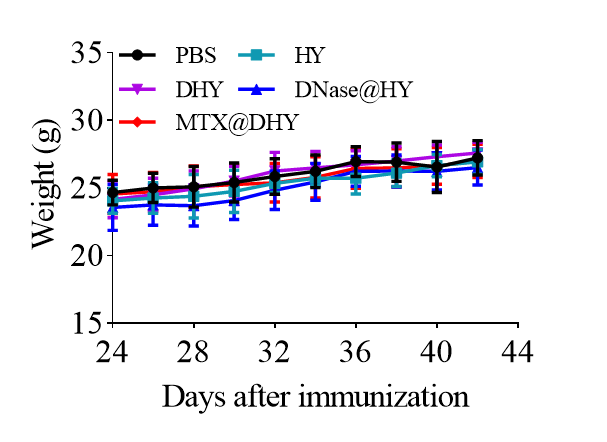


**Supplementary Figure 11.** Bodyweight of mice from different groups.
